# From Cognitive Maps to Cognitive Graphs

**DOI:** 10.1371/journal.pone.0112544

**Published:** 2014-11-12

**Authors:** Elizabeth R. Chrastil, William H. Warren

**Affiliations:** 1 Brown University, Cognitive, Linguistic, & Psychological Sciences, Providence, Rhode Island, United States of America; 2 Boston University, Department of Psychological and Brain Sciences, Center for Memory and Brain, Boston, Massachusetts, United States of America; University of Lethbridge, Canada

## Abstract

We investigate the structure of spatial knowledge that spontaneously develops during free exploration of a novel environment. We present evidence that this structure is similar to a labeled graph: a network of topological connections between places, labeled with local metric information. In contrast to route knowledge, we find that the most frequent routes and detours to target locations had not been traveled during learning. Contrary to purely topological knowledge, participants typically traveled the shortest metric distance to a target, rather than topologically equivalent but longer paths. The results are consistent with the proposal that people learn a labeled graph of their environment.

## Introduction

Imagine that you have arrived in a new city, and have spent a few hours wandering around the downtown. Now back at your hotel, you need some coffee. You remember seeing a coffee shop midway through your wanderings, but following your original route, with all its twists and turns, would be inefficient. You might not know the exact direction and distance of the coffee shop, but if you learned the network of streets, you could take a shorter path to your caffeine destination, even though you had not traveled that exact route before. Your knowledge of the network of streets, including places where they intersect, can be characterized as a graph of the downtown.

**Figure 1:**
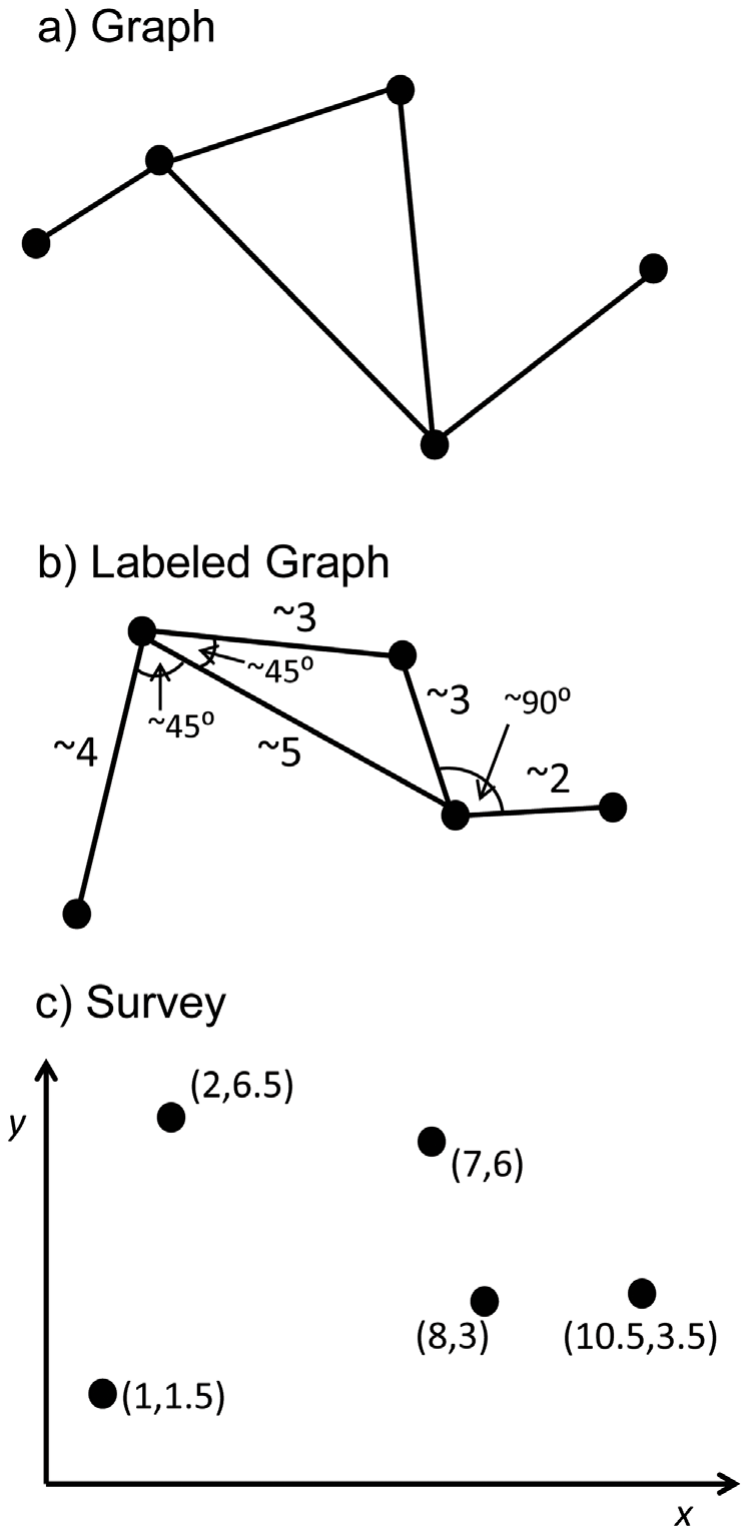
Illustration of three levels of spatial knowledge. a) Graph knowledge: purely topological graph of a network of place nodes (identifiable places, including junctions) linked by path edges (traversable paths between nodes), expressing the known connectivity of the environment. b) Labeled graph: incorporates local metric information about distances between known places (edge weights) and/or angles between known paths (node labels). Note that the topological structure of the connections between nodes is the same for a) and b). In the labeled graph, metric information may be coarse, contain biases, and is not globally consistent. c) Survey knowledge: configural map-like knowledge of environmental locations. Metric information is quite accurate and consistent throughout the region, embedded in a common coordinate system.

Several possible forms of spatial knowledge may underlie human navigation [1]. *Routes* are series of place-action associations, detailing a sequence of turns at each recognizable place. *Survey knowledge* (Figure 1c) is configural map-like knowledge of environmental locations, including the metric distances and directions between them. A cognitive map is commonly thought of as globally consistent survey knowledge with a common coordinate system, also known as a global metric embedding [2]. Such a Euclidean cognitive map might be built up by path integration during spatial learning [3], [4], and it would enable direct shortcuts between known locations.

What we will call *graph knowledge* is situated between route and survey knowledge. A purely topological graph of an environment (Figure 1a) consists of a network of place *nodes* (identifiable places, including junctions) linked by path *edges* (traversable paths between nodes) [5]. Thus, graph knowledge would express the known connectivity of the environment [6]–[11], enabling novel detours. In contrast, route knowledge does not accommodate multiple paths intersecting at a junction or multiple connections between the same locations, making detours difficult. On the other hand, survey knowledge contains metric information about distances and directions between environmental locations, allowing novel shortcuts [3].

A number of studies have cast doubt on a globally consistent Euclidean cognitive map [6], [12]–[16]. For example, distance estimates between locations frequently violate Euclidean axioms [15], [17]–[21], and the addition of turns [6], [22], intersections [23], or barriers [14] can increase the subjective distance of a route. A *labeled graph* (Figure 1b) can incorporate local metric information about distances between known places (edge weights) and/or angles between known paths (node labels), without being globally consistent. This local metric information might be coarser than survey knowledge, and may incorporate biases such as regularizing angles to 90°. Such augmented graph knowledge would be advantageous for finding efficient routes and detours, and would permit approximate shortcuts via local integration of information, without the complications of creating a global metric embedding necessary for survey knowledge.

The three basic forms of spatial knowledge described here—survey, graph, and route—form a hierarchy, in which the each level of the hierarchy encompasses the levels below it. Thus, if a navigator has complete survey knowledge, they also have access to graph and route information. Likewise, graph knowledge implies route information. Although all three levels of spatial knowledge may coexist, we propose that human navigation relies primarily on knowledge that can be characterized by a labeled graph.

Recently, we tested survey knowledge acquired during free exploration of a virtual hedge maze environment [24]. We probed survey knowledge using a novel shortcut task in which participants were asked to walk from the start object to the target object on a straight-line path. Accurate shortcuts depended on survey knowledge of the metric distances and directions between learned locations. But half of the participants were near chance (90°) and mean absolute shortcut errors were large, around 70°. A few participants may have learned approximate survey knowledge (12% had absolute errors around 20°), but there is little indication that this is the primary form of spatial knowledge acquired.

**Figure 2:**
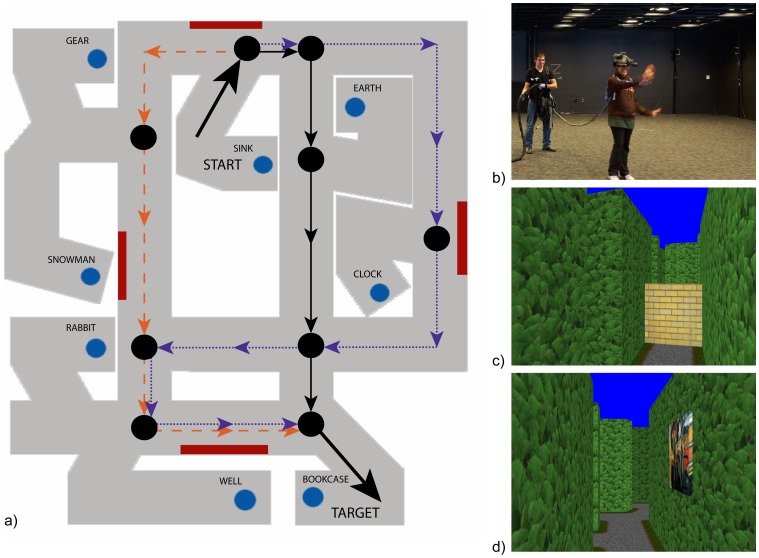
Virtual hedge maze. a) Overhead view of the maze, which participants never saw. Eight target objects (blue circles) and 4 landmark paintings (red rectangles) were placed in the maze. This figure shows an example of one of the object pairs: a direct trial from the sink (top) to the bookcase (bottom), illustrating the shortest path (solid black line), a topologically equivalent but longer path (dashed orange line), and a path that is both topologically and metrically longer (dotted purple line). Nodes in the paths are indicated by black circles. Thick black arrows indicate that all alternative paths started from the same location next to the sink, and ended by going into the branch hallway of the bookcase. b) View of the VENLab. c) View of landmark inside the maze. d) View of the barrier on a detour trial.

The aim of the present study is to experimentally distinguish route, graph, and labeled graph knowledge, using the same hedge maze environment. Although this experiment does not test survey knowledge, the results of our previous study, together with other evidence that spatial knowledge is not metrically Euclidean, imply that the underlying structure of spatial knowledge is likely to lie at a lower level in the hierarchy. Thus, our focus here is to distinguish graph knowledge from route knowledge, and a labeled graph from a purely topological graph. To achieve these aims, we recorded the routes participants travelled as they learned the maze, and then analyzed the direct paths or novel detours they took between learned locations. In the exploration phase, participants freely walked in the virtual environment to learn the locations of eight objects (Figure 2). In the test phase, their spatial knowledge was probed by starting participants at one of the objects, and asking them to walk through the hallways of the maze to a target object, taking the shortest route possible. Test trials included forced detours on nearly half the trials.

By comparing the paths traveled during the exploration and test phases, we are able to test the hypothesis that spatial knowledge is comprised of previously learned routes. Pure route knowledge predicts that the paths taken during test would have been previously traveled during exploration; significant use of novel paths would implicate more complex spatial knowledge, such as graph knowledge. We next distinguish labeled graph from topological graph knowledge by analyzing the path lengths during test. Labeled graph knowledge predicts that participants would select metrically shorter routes over topologically equivalent routes that have the same number of nodes but are metrically longer. In contrast, pure (unlabeled) graph knowledge predicts no preference in path selection between topological isomers. Our results indicate that the underlying structure of human spatial knowledge is consistent with a labeled graph.

## Methods

### Participants

34 volunteers (17 female) participated in the experiment and were paid for their time. One participant withdrew due to symptoms of simulator sickness, and one was excluded because they failed to find all of the objects during exploration. Sample size was determined by a power analysis to provide statistical power of 0.8, for an effect size of 0.5 and α = 0.05. All participants read and signed forms indicating their informed consent to participate in the experiment; this protocol was approved by the Brown University IRB (Protocol #0002990025).

### Equipment

The experiment was conducted in the VENLab, a 12 m×14 m ambulatory virtual reality facility (Figure 2b). Participants freely walked around the laboratory while wearing a head-mounted display (HMD) to view the virtual environment. Images were presented to participants in a Rockwell-Collins SR80A HMD (Cedar Rapids, IA) (63^o^ H×53^o^ V field of view, 1280×1024 pixels, 60 Hz frame rate). Head position and orientation were recorded using an InterSense IS900 (Billerica, MA) tracking system (50 ms latency, 60 Hz sampling rate, 1.5 mm RMS and 0.1° RMS accuracy). Participants responded by walking to target locations and pressing a button on a radio mouse. Images were generated on a Dell XPS graphics PC (Round Rock, TX) using Vizard software (WorldViz, Santa Barbara, CA) to render the images. Naturalistic evening sounds were presented over headphones to mask any auditory location or orientation cues.

### Environment

The 11 m×12 m virtual maze environment (Figure 2a,c) contained eight objects located in the terminal segment of branch hallways, so they were not visible from the main corridors. They were models of common objects, such as a sink or bookcase, scaled to be easily visible at eye level. In addition, four landmarks – familiar paintings by Monet, Dali, Magritte, van Gogh –appeared in constant locations on the walls of the maze in several main corridors, to aid orientation. The ground in each corridor was a gravel texture with a brown earthen and green grassy border.

### Procedure

Participants were informed that they would be traveling through hallways in a virtual hedge maze, and that the task was to find all of the objects and learn their locations. The virtual eye height corresponded to actual eye height of participants as measured by the head tracker.

#### Practice

Participants were given several minutes in a practice maze with a different layout from the test maze and which contained objects not used during the experiment, sufficient to correct the depth underestimation common in head-mounted displays [25].

#### Learning Phase

In the learning phase, participants were informed they would explore the environment for 10 minutes, during which time they should try to find all of the objects and learn their locations. They were guided to one of six start locations, and the experimental maze appeared. Participants explored the virtual environment by walking freely, providing them with normal visual, motor/proprioceptive, and vestibular information as well as the ability to make decisions during exploration.

#### Test Phase

Graph knowledge was tested using a shortest route task, in which the participant walked through the corridors of the maze from a starting object to the remembered location of a target object by the shortest route possible. Prerecorded instructions were presented to the participants over the headphones, and then repeated by the experimenter. Each trial began by wheeling the participant in a desert environment to the entrance of the branch hallway (where the branch hallway met the main hallway) containing the start object for that trial, approximately 1 meter from the object; the participant clicked the mouse, whereupon the maze and start object appeared. There were thus 8 start locations during the test phase. This procedure allowed participants to orient themselves while preventing more spatial learning during the test phase. The participant was instructed to walk to the start object, at which point the target object was named over the headphones, and they were given 30 seconds to reach the target location. Once the target was named, all objects, including the start and target objects, were replaced with red blocks during the remainder of the trial to avoid providing feedback and to ensure that participants did not simply search until they found the target; the four landmark paintings remained visible during the task. The trial ended when the participant clicked the mouse to indicate that they thought they had reached the target location, or 30 seconds elapsed. The maze then disappeared, and the experimenter wheeled the participant to the starting location of the next trial, taking a circuitous route to prevent participants from learning more about object locations between trials. Position and orientation were recorded throughout the trial, with the location of the final mouse click serving as the endpoint for the trial.

Participants completed two practice trials on object pairs not used during the test phase, followed by 40 test trials. Crucially, 40% of the test trials were “detour” trials, in which one of the shortest paths was blocked with a barrier, and participants were instructed to find a novel route to the desired object (Figure 2d). Because the barrier often required additional time and distance to travel around it, participants were given 15 additional seconds on detour trials, although they were not informed of the additional time. There were 8 pairs of starting and target objects, with 5 trials each; 3 of them were direct trials and 2 were detour trials. All trials were presented in a random order with the exception that an object pair did not repeat back-to-back.

#### Follow-up tests

Finally, individual differences in spatial ability could affect the navigational strategies used in this task [26]. Participants performed several standard tests of spatial ability. First, they completed (a) the Santa Barbara Sense of Direction Scale (SBSOD), in which lower scores indicate better sense of direction [27]. They also filled out (b) a questionnaire including report of current and past video game use, which has been shown to be a factor in virtual navigation [28]. They completed (c) the Road Map Test (RMT) [29], [30], in which participants report the direction of each turn (left or right) in a route drawn on a city map, modified to have a 20-second time limit. Finally, participants were given (d) the Perspective-Taking Test (PTSOT) [31], in which they view a 2D array of objects on a page, and indicate their directions from different imagined viewpoints. The Road Map Test and Perspective-Taking Tests gauge a navigator's ability to process location and direction information from different perspectives, which could be important for acquiring graph knowledge during exploration or for orienting within the maze during the test phase.

### Analysis

Analysis was performed using MatLab (MathWorks), and SPSS (IBM) software. The *proportion of correct target locations* was the primary dependent measure, where a trial was considered correct if the participant ended the trial anywhere in the branch hallway of the target object. In addition to the overall proportion correct, direct and detour trials were examined separately, using a within-subjects design where appropriate. The chance level for proportion correct was defined as ending a trial at the location of any of the 8 target objects, or 1/8 = 0.125.

Paths were analyzed using a set of invisible portals placed at all decision points in the maze, which recorded when a participant crossed between different hallway sections. This setup allowed for direct comparison between a series of portals traveled on a test trial and a specified series of portals (e.g. the shortest path or a detour), or a sequence of portals during the learning phase. Most analysis was performed only on correct trials. We first analyzed paths for reliance on route knowledge during the test phase. Pure route knowledge predicts that the path used during test would match one of the routes taken during exploration, with no novel paths. If the sequence on a test trial matched any sequence from the learning phase, in either the forward or backward direction, it was counted as a match, and thus indicated that participants relied on familiar routes during the test phase.

We next examined whether participants took the shortest path to the target. The labeled graph hypothesis predicts that the shortest path will be taken more frequently than other alternatives. The (unlabeled) topological graph hypothesis predicts that the path with the fewest number of nodes will be selected, without consideration of path length. For direct trials, if two paths were equally long (e.g. traveling either direction around a rectangular loop), they were both counted as the shortest path. For detour trials, a path counted as the shortest only if the participant started on the normally shortest path to the target before encountering the barrier, and then took the shortest detour from that point. Alternative paths with the same number of nodes in the graph (topologically equivalent but longer distance) were analyzed in a similar manner. Bends in a hallway that were not choice points (e.g. the elbow turn in upper right corner of maze, Figure 1a) were not counted as nodes, since there was only a direction change. If there were multiple topologically equivalent alternative paths, they were treated as one alternative path, providing a more stringent test. The proportion of trials in which the shortest path was taken was compared to the proportion of trials in which any alternative path was taken, using paired t-tests. Participants who did not have any correct trials on the object pairs in question were not included in the t-tests.

Finally, to investigate the factors that made some object pairs more difficult than others, we computed Pearson correlations between the proportion correct on each pair and (a) the path length between the start and target object, (b) the number of edges in the graph between the objects, (c) the Euclidean distance between the objects, (d) the number of turns between the objects (including turns that were not choice points), (e) the number of times participants took the same route between objects during exploration, and (f) the number of times participants visited the target object during exploration.

In addition to the navigation and exploration measures, we examined the relationship between performance and spatial ability. We computed Pearson correlations between the four spatial abilities tests and the following three outcome measures: (1) the proportion of correct trials (direct and detour), (2) the proportion of trials in which the participant took a novel route (direct and detour), (3) the proportion of trials in which the shortest path was taken to the target (direct and detour).

## Results

**Figure 3:**
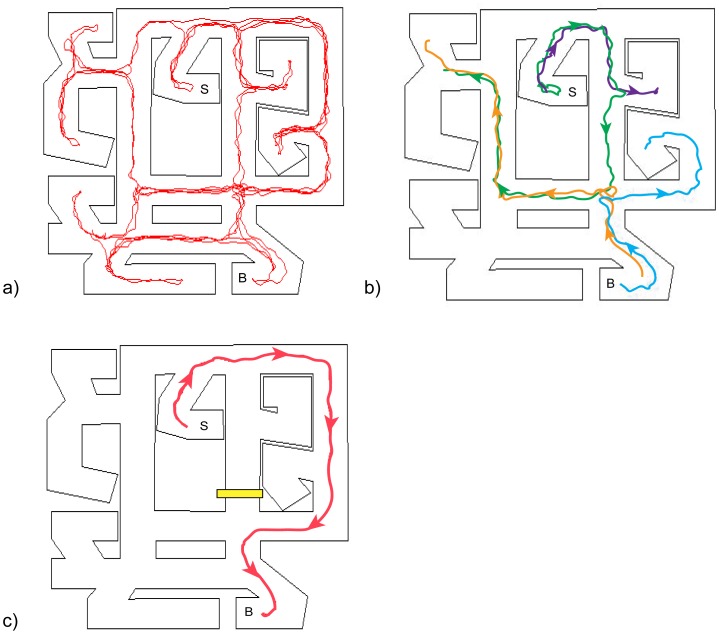
Novel routes taken during test. An example of a trial starting at the sink (S) and ending at the bookcase (B). a) All paths taken by a representative participant during the 10-minute exploration. b) Those exploration paths that start either from the sink (green, purple) or from the bookcase (orange, blue). c) A novel detour taken during test. The participant never traveled on that path or the reverse path during exploration.

**Figure 4:**
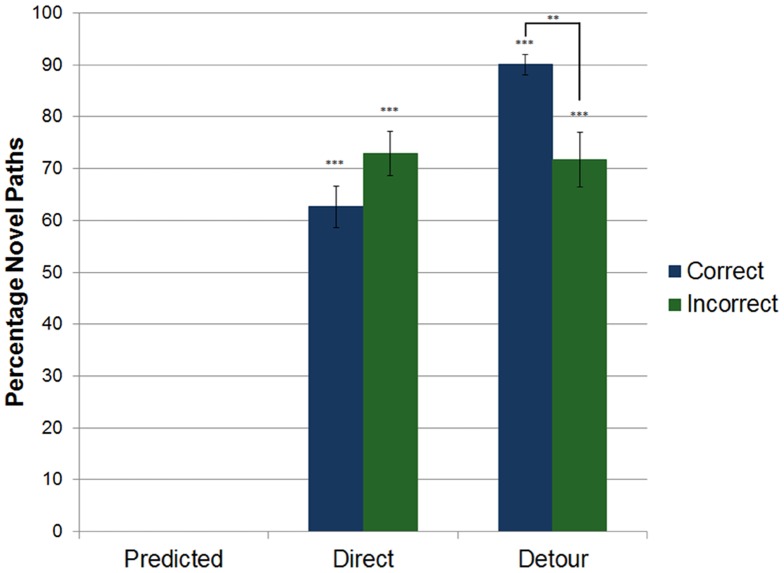
Percentage of trials in which a novel path was taken. Route knowledge predicts that none of the paths would be novel. Error bars indicate standard error of the mean. *** indicates p<0.001, 1-sample t-test with 0.

Overall, participants successfully reached the target within the time limit on 53.3% (SD = 31.2) of “direct” trials and 59.8% (SD = 28.6) of “detour” trials, both far above the chance level of 12.5% (direct: t_31_ = 7.401, p<0.001, Cohen's d = 1.308; detour: t_31_ = 9.339, p<0.001, Cohen's d = 1.651). While the error rate may appear high, we note that many of the errors were due to 4 participants who performed at chance; the other participants were successful on 59.7% of direct trials and 66.3% of detour trials. To test whether participants acquired graph knowledge of the maze, rather than simply route knowledge, we compared the paths taken during the test phase with the routes traveled during exploration. Pure route knowledge would only enable participants to use familiar routes to reach the target object, which they had previously traversed during exploration, and thus predicts that no novel routes would be taken on test trials. On successful trials, 62.6% of the direct routes were novel, and fully 90.1% of detour trials were novel (see Figure 3). One-sample t-tests confirmed that the proportion of novel routes was greater than 0 for both direct (t_31_ = 15.710, p<0.001, Cohen's d = 2.777) and detour trials (t_31_ = 44.786, p<0.001, Cohen's d = 7.917) (Figure 4). These findings suggest that when participants learned the spatial layout of the maze, they did not simply acquire knowledge of specific routes between locations in the maze, but instead learned a graph of connections between places, which could be recombined in order to generate novel paths and detours.

For incorrect trials, a novel route was taken on 73.0% of direct trials, which did not differ significantly from correct direct trials (t_30_ = −1.553, p = 0.131) (Figure 4). In contrast, for incorrect detour trials, the proportion of novel routes, 71.7%, was significantly lower than on correct detour trials (t_25_ = 3.407, p = 0.002). This result suggests that when faced with a detour, participants who reverted to familiar routes were less successful than those who generated a novel path to the target location.

We observed that particular “foils” were often selected instead of the correct target, providing insight into unsuccessful navigation. The most common navigational confusion occurred between the gear/snowman group and the rabbit/well group, which had similar branch hallway shapes and relationships within the graph. On trials starting at the snowman with the rabbit as the target, participants selected the gear on a large percentage of trials, likely based on the shape of the branch hallway, without ever entering the main hallways. Most of the foils either had similar branch hallway shapes or were near each other, such as the sink and the earth. Including the most common foil in addition to the correct target accounts for 69.6% of direct trials and 77.0% of detour trials; these numbers increase to 74.7% and 84.5%, respectively, when the two most common foils are included. Thus, participant errors were fairly predictable, and suggest that visually similar target branch hallways are sometimes misassigned to the wrong nodes in the graph structure.

We next tested whether the graph knowledge acquired by participants was consistent with a labeled graph, such that some local metric information was also learned. We examined the number of trials in which participants took the shortest available path to the target object. On successful trials, participants took the shortest path on 64.3% of the direct trials, and 73.3% of detour trials. We compared these proportions against chance, defined by the number of alternative paths that could result in successfully reaching the target without doubling back, repeated segments, or self-intersection. Participants took the shortest path more often than expected by chance in both direct (t_31_ = 9.592, p<0.001, Cohen's d = 1.696) and detour (t_31_ = 12.563, p<0.001, Cohen's d = 2.221) trials. This result clearly suggests that information about path length was used to select the path to the target.

However, there are at least two possible measures of path length, which are highly correlated: *metric distance*, the path length measured in meters, and *topological distance*, the number of nodes or edges on the path. To compare a topological graph with a labeled graph, we examined cases in which there were alternative paths between the start and target objects that possessed the same number of nodes as the shortest metric path. If participants took the shortest metric path more often than alternative paths having the same topological length, then this result would strongly imply some metric knowledge. Of the 8 object pairs, 2 had at least one alternative path with equivalent topological length on direct trials, and 3 others had such alternative paths on detour trials. On all five of these pairs, participants took the shortest metric path more often than they took all of the topologically equivalent paths combined (Table 1). Overall, they took the shortest path on 63.0% of correct trials, and the topologically equivalent path on 21.6% of correct trials for these pairs. On 3 of the 5 pairs, the shortest path was taken significantly more often than the equivalent alternatives (p<0.01 or better). A fourth pair was marginally significant, while the fifth pair was not statistically different from its two alternatives. Furthermore, for the marginal pair (pair number 4, direct) the alternative paths had fewer total turns and nodes than the metrically shortest path, yet participants took the shortest path more often. In addition, the number of participants who took these shortest paths at least once ranged from 37.5–50% of the total. These findings imply that participants were sensitive to metric distance information, corresponding to edge weights in a labeled graph.

**Table 1:** Proportion of correct trials in which participants took the shortest path compared with alternatives with an equal number of nodes in the graph.

Object pair (direct/detour) (# of alternatives)	Proportion Taking Shortest Path	Proportion Taking Any Topologically Equal Alternative	N (# of participants who took the shortest route at least once)	p-value
1 (direct) (2)	0.458	0.451	24 (13)	0.970
2 (detour) (1)	0.875	0.000	16 (14)	<0.001
3 (detour) (1)	0.767	0.067	15 (12)	<0.001
4 (direct) (1)	0.627	0.310	21 (16)	0.074
7 (detour) (1)	0.790	0.132	19 (16)	<0.001

Some participants did not have any correct trials for the certain object pairs and were excluded from analysis; N indicates the number of participants who successfully reached the target in each condition and were included in each analysis.

We also observed that some object pairs were more difficult than others, with success on direct trials ranging from 41% to 71%. We examined the correlation between percent success on the 8 pairs and several measures of the relationship between the start and target objects that might affect difficulty. Of these potential factors, only the number of turns between the objects (including turns that were not at choice points) correlated significantly (*r*_6_ = −0.847, p = 0.008); whereas the Euclidean distance between them, the metric and topological path lengths, and route familiarity from exploration did not. This result demonstrates that route-finding difficulty increases with the number of turn angles that must be recovered during route selection, and suggests that turns should be included as labeled nodes in the graph, even when they are not choice points. Such local metric knowledge would enable approximate shortcuts without a global Euclidean map. This finding is consistent with studies demonstrating that spatial errors are frequently made when navigators must imagine a change in direction [32].

Finally, we examined the relationship between spatial abilities and test performance. The SBSOD self-report scale was marginally correlated with performance on direct trials (*r*_30_ = −0.349, p = 0.050), but not for detour trials (*r*_30_ = −0.274, p = 0.129). No other significant relationships were observed (all p>0.1). Although there were individual differences in performance, they do not appear related to these spatial abilities measures.

## Discussion

In this study, we investigated the primary structure of spatial knowledge that spontaneously develops during free exploration of a novel environment. The results are consistent with the proposal that people learn a labeled graph of their environment: a network of topological connections between places, labeled with local metric information. In contrast to route knowledge, we found that the most frequent routes and detours to target locations in the test phase had not been traveled during learning. The preferred paths were typically the shortest metric distance to a target, rather than topologically equivalent but longer paths, contrary to purely topological knowledge. Incorrect trials were associated with increased reliance on route knowledge and selection of foil objects, whereas better performance was strongly correlated with a smaller number of turns to the target.

The findings of this experiment suggest that a labeled graph is the most appropriate level in the hierarchy to characterize the primary form of spatial knowledge used during active navigation, at least in a newly-learned environment. Route knowledge may be described as a subgraph in this larger structure, whereas the full graph enables new routes and detours. For comparison with survey knowledge, we previously used this same maze to test survey learning in a separate group of participants [24]. This test required participants to take direct straight-line shortcuts between learned locations. In contrast to the large errors and 50% of participants near chance in that study, only 10% of participants in the present study performed near the chance level, half were successful in route-finding on about 60% or more trials, and about 40% were able to take the shortest routes. Despite differences in administering and scoring the two tasks, this pattern of results implies that the majority of participants acquired graph knowledge, but not survey knowledge, when freely exploring the same environment. Furthermore, humans and rodents tend to explore edges and landmarks in novel environments, rather than open space [33], [34]. This strategy is efficient for building a graph, but not for complete survey knowledge. Although we cannot rule out the possibility that some participants acquired survey knowledge in the present study, our results are most consistent with the hypothesis that primary spatial knowledge is aptly characterized as a labeled graph.

The relationship between allocentric (world-centered) and egocentric (viewer-centered) navigation must also be considered with regard to these levels of spatial knowledge. Route knowledge has typically been considered to be egocentric, and survey knowledge to be allocentric. Graph knowledge may be intermediate between these two perspectives, and might be used flexibly from either an egocentric or allocentric viewpoint [10]. It is possible that the most successful navigators were those who were able to translate between perspectives [26], [35].

Finally, we offer some caveats regarding the limitations of these findings. Although the task demands were similar to those encountered in everyday navigation tasks, the environment had a maze-like structure and participants were given a limited time to learn it. With additional experience or a more open environment, navigators might learn specific routes or acquire more accurate survey knowledge. Nevertheless, the present results show that participants do not first simply acquire route knowledge of a novel environment, for they were able to take novel paths early in the test phase. Neural evidence indicates that brain areas supporting habitual route learning are active at a later stage of learning [e.g. 36,37], consistent with the idea that navigators rely on different forms of spatial knowledge at different stages of learning and for different purposes [38]. In addition, our analyses were primarily conducted on correct trials, thus many of our conclusions are limited to instances in which participants had learned enough about the environment to correctly reach the goal location.

Nevertheless, the present findings offer the first empirical evidence for a labeled graph as an appropriate description of the primary spatial knowledge used to guide active navigation. Such a “cognitive graph” is sufficient to account for apparently Euclidean behavior like shortcuts and detours without requiring globally consistent metric maps.
